# Atypical presentation of giant cell arteritis in a patient with vertebrobasilar stroke: A case report

**DOI:** 10.1097/MD.0000000000016737

**Published:** 2019-08-09

**Authors:** Ahmed Mohamed Elhfnawy, Michael Bieber, Mira Schliesser, Peter Kraft

**Affiliations:** a Department of Neurology, University Hospital of Würzburg, Würzburg, Germany; b Department of Neurology, University Hospital of Alexandria, Alexandria, Egypt; c Department of Neurology, University Hospital of Essen, Essen, Germany.

**Keywords:** atypical, bilateral halo sign, giant cell arteritis, stroke, temporal artery, ultrasound, vertebrobasilar

## Abstract

**Rationale:**

Giant cell arteritis (GCA) is known to present with typical manifestations like temporal headache and visual abnormalities. However, several cases with atypical manifestations were reported. Stroke occurs in 3% to 7% of patients with GCA.

**Patient concerns:**

A 67-year-old male patient with known hypertension presented with somnolence, disorientation and mild bilateral limb ataxia. The magnetic resonance imaging showed multiple acute infarctions in the territory of the vertebrobasilar system with occlusion of the left vertebral artery.

**Diagnosis:**

Ten months later, during a routine neurovascular follow-up, recanalization of the left vertebral artery was observed and a hypoechoic concentric “halo” sign around both vertebral arteries, mainly on the left side was evident. On further examination of the superficial temporal artery, a hypoechoic concentric “halo” sign was also found, which—along with increased inflammatory markers—raised suspicion about GCA. Classical GCA features like headache, temporal tenderness or amaurosis fugax were not present. Repeated in-depth diagnostic work-up including 48 hours Holter-ECG did not reveal another stroke etiology.

**Interventions:**

Intravenous Methylprednisolone 250 mg/d was immediately started and after 6 days the dose was tapered to 80 mg/d. The patient was discharged on a tapering scheme with the recommendation to start azathioprine. Additionally, we placed the patient on acetylsalicylic acid 100 mg/d and clopidogrel 75 mg/d. However, the patient was not compliant to treatment; he stopped prednisolone early and did not start azathioprine.

**Outcomes:**

The inflammatory markers were markedly reduced at the beginning of the treatment. After stopping the immunosuppressive medications, the inflammatory markers were once again increased. Three months later, the patient developed bilateral middle cerebral artery and right occipital lobe infarctions.

**Lessons:**

In patients with cryptogenic vertebrobasilar strokes, GCA may be considered in the differential diagnosis, especially if the inflammatory markers are increased.

## 1 Introduction

Giant cell arteritis (GCA) is the most common systemic vasculitis in Northern Europeans aged ≥50 years with an annual incidence of 15–33/100.000.^[1]^ The classical manifestations of patients with GCA are headache, temporal tenderness, anterior ischemic optic neuropathy or amaurosis fugax up to permanent visual loss, thickening and tortuosity of the temporal artery, polymyalgia rheumatica, fever, and increased erythrocyte sedimentation rate (ESR) >50 mm in the 1st hour.^[2]^ However, atypical cases without headache or visual manifestations have been frequently reported.^[2–6]^ Stroke affects about 3% to 7% of patients with GCA.^[7–9]^ Whether GCA should be routinely investigated in stroke patients remains a matter of discussion.

## 2 Case presentation

**Figure 1:**
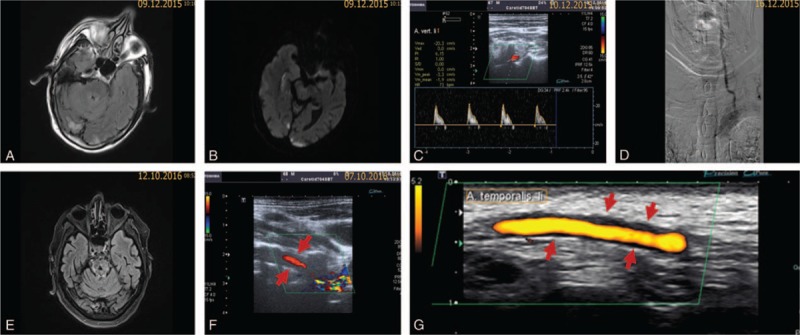
Fluid-attenuated inversion recovery **(A)** and Diffusion-weighted imaging **(B)** in 12/2015 showed multiple small acute infarctions in the vertebrobasilar territory. Duplex ultrasound of the left vertebral artery showed occlusion signal denoting distal occlusion **(C)**, which was confirmed in the DSA **(D)**. FLAIR in 10/2016 showed a lacunar cavity in the pons **(E)**, which was not present in the initial MRI images. Duplex ultrasound in 10/2016 showed recanalization and “halo” sign (red arrows) around the left vertebral artery **(F)**. Ultrasound of the left superficial temporal artery in 10/2016 showed “halo” sign (red arrows) supporting the diagnosis **(G)**.

A 67-year-old male was admitted to our stroke unit in December 2015 because of multiple bilateral small infarctions in the distribution of the vertebrobasilar territory, involving both cerebellar hemispheres, both occipital lobes and right thalamus (Fig. 1A+B). The patient was known to suffer from hypertension and mild dementia as well as rheumatoid arthritis. He was on a treatment with Methotrexate 10 mg once per week and folic acid 5 mg once per day. The patient was known to smoke 1 pack cigarettes per day with around 50 pack years. On examination, the patient was somnolent and disoriented to time and place. Minimal dysmetria on both sides was revealed during finger-to-nose test and the gait was wide based. National Institute of Health Stroke Scale (NIHSS) on admission was 3 and C—reactive protein (CRP) on admission was 0.47 mg/dL (reference value ≤0.5 mg/dL). Extensive diagnostic work-up including 48 hours Holter ECG, transthoracic (TTE) and transesophageal echocardiography (TEE) revealed no specific abnormalities. The neurovascular imaging using ultrasound and digital subtraction angiography (DSA) revealed occlusion of the left internal carotid bulb, occlusion of the left vertebral artery (Fig. 1C+B), filiform stenosis of the right vertebral artery at the level of dural penetration and 70% stenosis of the right internal carotid artery. The superficial temporal arteries have not been examined. After detailed discussions and presenting the patient in our neurovascular board, we placed the patient on acetylsalicylic acid 100 mg/d, clopidogrel 75 mg/d and simvastatin 40 mg/d. Since the patient's rheumatoid arthritis was in complete remission, we stopped methotrexate. On discharge, the modified Rankin Scale (mRS) was 3.

Ten months later, the patient presented in our neurovascular outpatient clinic for a routine follow-up. Meanwhile, the mRS was still 3 and the patient did not develop any new transient or permanent neurological deficits. On ultrasound examination, recanalization of the left vertebral artery was found, yet a hypoechoic concentric “halo” sign around both vertebral arteries mainly on the left side was observed (Fig. 1F). On further examination of the superficial temporal artery, a hypoechoic concentric “halo” sign was also present (Fig. 1G). CRP and ESR/1st hour were 4.77 mg/dL and 62 mm (reference value <34 mm according to the formula age in years/2)^[10]^, respectively. Headache, visual problems or any manifestations related to rheumatoid arthritis have been denied. On palpation, tenderness of the temporal arteries was not present. On brain magnetic resonance imaging (MRI), the following findings were present:

a) acute (asymptomatic) infarction in the left postcentral gyrus,
b) progression of the white matter lesions, and
c) lacunar pons infarction (Fig. 1E), which was not seen in the previous MRI.

Extensive laboratory work-up for various immunological and rheumatological diseases as well as cerebrospinal fluid (CSF) examination revealed no specific abnormality. GCA was suspected and the patient received intravenous Methylprednisolone 250 mg/d over 6 days and then tapered to 80 mg/d. The patient was discharged on a tapering scheme with the recommendation to start azathioprine. The inflammatory markers were markedly reduced at the beginning of the treatment. In addition, we again placed the patient on acetylsalicylic acid 100 mg/d and clopidogrel 75 mg/d. However, the patient was not compliant to treatment; he stopped prednisolone early and did not start azathioprine. One month later, the inflammatory markers were once again increased with a CRP-value of 6.43 mg/dL and an ESR-value of 63 mm in the first hour, respectively. Three months later, the patient was admitted to another hospital because of bilateral middle cerebral artery and right occipital lobe infarctions with mRS of 4. Again, tenderness of the temporal arteries was not present. The CRP-value was 9.2 mg/dL (reference value ≤0.5 mg/dL). Repeated cardiac work-up including TTE, TEE, and 24 hours Holter ECG again showed no source of embolism. The patient refused to undergo further diagnostic work-up including a biopsy from the superficial temporal artery. The patient provided a written informed consent for the publication of this case report.

## 3 Discussion

We present a patient with delayed diagnosis of GCA-related ischemic stroke with atypical presentation. The first admission to our hospital occurred because of stroke in the vertebrobasilar territory with normal inflammatory markers. The patient was known to suffer from rheumatoid arthritis and was on methotrexate. Methotrexate was stopped. Thereafter, the patient suffered at least 2 further ischemic stroke events; both were associated with increased inflammatory markers. In addition, the white matter lesions progressed and new lacunar brainstem lesions were shown in a second brain MRI. According to the American College of Rheumatology (ACR), the diagnosis of GCA is based on the presence of 3 out of the following 5 criteria: age ≥50 years at disease onset, new onset of localized headache, ESR ≥50 mm/hour, tenderness or decreased pulse of the temporal artery, and a biopsy from the temporal artery showing inflammatory cells with or without multinucleated giant cells.^[11]^ In our patient, taking into account that biopsy of the temporal artery was not performed, only 2 of these 5 criteria were met. However, ultrasound examination of the vertebral and superficial temporal artery showed a “halo” sign. In recent studies, ultrasound examination of the superficial temporal artery was shown to have a specificity of 81% to 96% for diagnosing GCA.^[12,13]^ The ACR criteria, which were introduced in 1991, do not take “halo” sign into consideration. Actually, this sign was first described in 1995 by Schmidt et al, opening the door for a major successive body of literature.^[14]^ Since then, ultrasound has emerged as an important tool in the diagnosis of GCA.^[15]^ In patients with typical clinical manifestations and vascular edema around the temporal artery in ultrasound (“halo” sign), further invasive investigations like temporal artery biopsy may be omitted.^[15]^ The diagnosis of GCA relying on temporal artery biopsy can yield false-negative results in up to 60% of cases.^[16]^ These can be ascribed to a delay in sampling of the biopsy especially after starting corticosteroid treatment or due to sampling of a non-vascular or a non-inflamed segment.^[2,16]^ Because of the bad neurological condition with mRS of 4, the patient refused to undergo a temporal artery biopsy, so that the ACR-criteria were not fulfilled.

Several studies examined the association between GCA and stroke.^[7,17–19]^ A recent retrospective study found that the vertebrobasilar territory was involved in 73% of stroke patients related to GCA, in comparison to only 15% to 20% of patients with atherosclerosis-related strokes.^[17]^ Our patient experienced several ischemic attacks in the posterior and anterior circulation.

Thrombo-inflammation has emerged as a new pathophysiological concept connecting thrombus formation with inflammation.^[20]^ After a plethora of preclinical murine studies, the first small clinical trials proved efficacy of immune-modulating drugs in stroke.^[21,22]^ In patients with GCA, the pathophysiology of stroke seems to be related to inflammatory mechanisms rather than thromboembolism.^[17]^ In fact, we cannot conclude whether the recurrent strokes in our patient were related to atherosclerosis, vasculitis, or even cardioembolism. However, given the ultrasound finding (“halo” sign around both vertebral and temporal arteries) and the spontaneous recanalization of the left vertebral artery, we assume that at least the vertebrobasilar strokes might have been related to vasculitic processes. Furthermore, the development of lacunar pontine infarction raises suspicion about an ongoing vasculitic process. Rheumatoid arthritis-associated cerebral vasculitis seems to be unlikely in our patients for the following reasons:

1. the absence of classical painful joint swelling in association with the increased inflammatory markers and
2. The presence of “halo” sign of the vertebral and the superficial temporal artery, which is not known to occur in patients with rheumatoid arthritis.

A retrospective cohort study from Spain found an increased risk of stroke, mainly in the vertebrobasilar territory in the first 4 weeks after GCA diagnosis.^[7]^ In our patient, GCA was diagnosed 10 months after vertebrobasilar stroke. On initial presentation, the patient did not have increased inflammatory markers. However, this is not an exclusion criterion and has been previously described in GCA-related stroke patients.^[17]^ A recent population-based study from Canada found the risk of stroke to be 2 times higher and the risk of myocardial infarction 3 times higher among GCA patients compared to the general population. The risk is especially increased in the first year after diagnosis.^[19]^

Epidemiological studies addressing the prevalence of GCA in stroke patients are sparse. In a study from Spain, 5 out of 1237 (0.4%) patients with stroke were found to have GCA.^[18]^ In this study, the authors screened their patients for the presence of “halo” sign around the vertebral artery. Since this Spanish cohort included patients with stroke, either in the carotid or the vertebrobasilar territory, and the vertebrobasilar territory is known to be involved in 73% of stroke patients related to GCA,^[17]^ it might be postulated that the incidence of GCA among patients with vertebrobasilar stroke might be higher. From the authors’ point of view, many GCA-related stroke patients could have been missed in the aforementioned study, because ultrasound examination of the superficial temporal artery is the standardized examination for GCA and the superficial temporal artery is the most commonly affected artery in patients with GCA.^[23]^ Furthermore, the incidence in northern Europe seems to be at least 2 times higher than parts of southern Europe.^[24,25]^

## 4 Conclusion

Increased inflammatory markers in stroke patients may be considered as a warning sign requiring further simple investigations like ultrasound of the superficial temporal artery. Ultrasound examination of the temporal arteries could be suggested in a screening study in patients with cryptogenic vertebrobasilar strokes, even in the absence of inflammatory markers or other criteria for GCA. Nevertheless, it remains a subject for future clinical research, whether this diagnostic strategy has the necessary accuracy, sensitivity, and specificity to improve the detection of GCA-related stroke patients.

## Author contributions

**Conceptualization:** Ahmed Mohamed Elhfnawy, Michael Bieber, Mira Schliesser, Peter Kraft.

**Data curation:** Ahmed Mohamed Elhfnawy, Michael Bieber, Mira Schliesser, Peter Kraft.

**Methodology:** Ahmed Mohamed Elhfnawy.

**Project administration:** Ahmed Mohamed Elhfnawy.

**Software:** Michael Bieber.

**Supervision:** Mira Schliesser, Peter Kraft.

**Writing – original draft:** Ahmed Mohamed Elhfnawy.

**Writing – review & editing:** Michael Bieber, Mira Schliesser, Peter Kraft.

Ahmed Mohamed Elhfnawy orcid: 0000-0001-9800-7830.
