# Atypical presentation of giant cell arteritis in a patient with vertebrobasilar stroke: A case report: Erratum

**DOI:** 10.1097/MD.0000000000017086

**Published:** 2019-08-30

In the article, “Atypical presentation of giant cell arteritis in a patient with vertebrobasilar stroke: A case report”,^[1]^ which appears in Volume 98, Issue 32 of *Medicine*, Dr. Peter Kraft's affiliations should appear as Department of Neurology, University Hospital of Würzburg, Würzburg, Germany; and Klinikum Main-Spessart Lohr, Lohr, Germany.
